# Metatranscriptomic analysis of lignocellulolytic microbial communities involved in high-solids decomposition of rice straw

**DOI:** 10.1186/s13068-014-0180-0

**Published:** 2014-12-31

**Authors:** Christopher W Simmons, Amitha P Reddy, Patrik D’haeseleer, Jane Khudyakov, Konstantinos Billis, Amrita Pati, Blake A Simmons, Steven W Singer, Michael P Thelen, Jean S VanderGheynst

**Affiliations:** Joint BioEnergy Institute, Emeryville, CA 94608 USA; Department of Food Science and Technology, University of California, Davis, CA 95616 USA; Department of Biological and Agricultural Engineering, University of California, One Shields Ave, Davis, CA 95616 USA; Physical and Life Sciences Directorate, Lawrence Livermore National Laboratory, Livermore, CA 94551 USA; Joint Genome Institute, Walnut Creek, CA 94598 USA; Biological and Materials Science Center, Sandia National Laboratories, Livermore, CA 94551 USA; Earth Sciences Division, Lawrence Berkeley National Laboratory, Berkeley, CA 94720 USA

**Keywords:** Lignocellulose deconstruction, Solid-state culture, Microbial communities, Biofuels, Cellulase, Glycoside hydrolase family 48, Carbohydrate binding module family 2, Carbohydrate binding module family 33

## Abstract

**Background:**

New lignocellulolytic enzymes are needed that maintain optimal activity under the harsh conditions present during industrial enzymatic deconstruction of biomass, including high temperatures, the absence of free water, and the presence of inhibitors from the biomass. Enriching lignocellulolytic microbial communities under these conditions provides a source of microorganisms that may yield robust lignocellulolytic enzymes tolerant to the extreme conditions needed to improve the throughput and efficiency of biomass enzymatic deconstruction. Identification of promising enzymes from these systems is challenging due to complex substrate-enzyme interactions and requirements to assay for activity. In this study, metatranscriptomes from compost-derived microbial communities enriched on rice straw under thermophilic and mesophilic conditions were sequenced and analyzed to identify lignocellulolytic enzymes overexpressed under thermophilic conditions. To determine differential gene expression across mesophilic and thermophilic treatments, a method was developed which pooled gene expression by functional category, as indicated by Pfam annotations, since microbial communities performing similar tasks are likely to have overlapping functions even if they share no specific genes.

**Results:**

Differential expression analysis identified enzymes from glycoside hydrolase family 48, carbohydrate binding module family 2, and carbohydrate binding module family 33 domains as significantly overexpressed in the thermophilic community. Overexpression of these protein families in the thermophilic community resulted from expression of a small number of genes not currently represented in any protein database. Genes in overexpressed protein families were predominantly expressed by a single Actinobacteria genus, *Micromonospora*.

**Conclusions:**

Coupling measurements of deconstructive activity with comparative analyses to identify overexpressed enzymes in lignocellulolytic communities provides a targeted approach for discovery of candidate enzymes for more efficient biomass deconstruction. Glycoside hydrolase family 48 cellulases and carbohydrate binding module family 33 polysaccharide monooxygenases with carbohydrate binding module family 2 domains may improve saccharification of lignocellulosic biomass under high-temperature and low moisture conditions relevant to industrial biofuel production.

## Background

Bioconversion of lignocellulosic biomass into liquid fuels is a potential strategy for offsetting the use of fossil fuels and reducing carbon emissions [1]. Such bioconversion requires that polysaccharides within lignocellulose be digested into fermentable monosaccharides. While enzymatic hydrolysis using lignocellulolytic enzymes is a standard approach for digestion, enzymes must be tolerant to several potentially inhibitory conditions including high temperatures associated with biomass pretreatment or heating to decrease the viscosity and required mixing energy of the biomass suspension [2,3] and high-solids environments necessary for minimizing water use [4]. Moreover, inhibitors derived from the biomass itself present additional challenges for deconstructive enzymes [5]. Given these constraints and the costs of enzymes, using enzymes optimized for industrial deconstruction processes that maintain activity under harsh industrial conditions is economically important [6].

Prior work has considered deconstructive microbial communities enriched on biofuel feedstocks as a source of useful enzymes for hydrolyzing lignocellulose [7]. In particular, metagenomic analysis of enriched communities has identified genes that potentially encode robust cellulases that are active in a high-temperature, high-solids environment [8]. While the representation of certain glycoside hydrolases in enriched deconstructive microbial communities provides promising gene targets, it offers no insight into whether communities actually express these genes. Metatranscriptomic analysis of these communities may refine the array of target genes identified via metagenomics by highlighting deconstructive enzymes expressed within the community. This is particularly important for deconstructive microbial communities, where digestion of the various components of lignocellulose may result from enzymes spanning different species, some of which may not be abundant within the community and thus not readily identified through metagenome analysis.

In this study, microbial communities from green waste compost were enriched on rice straw as a sole carbon source under high-solids loading conditions to select for target deconstructive microorganisms. Rice straw is an appealing biofuels feedstock since it is rich in lignocellulose and is generated in great quantities as a byproduct of rice production [9]. Green waste compost was selected as the inoculum for enrichment cultures, as it is generated under conditions similar to those that may be used in industrial bioconversion processes. These conditions include high temperatures, limited moisture, and the use of lignocellulose as the primary substrate. As a result, compost microbial communities are likely sources of deconstructive microorganisms and enzymes that may perform well under industrial conditions. High-solids enrichment cultures were conducted on un-pretreated rice straw under mesophilic or thermophilic conditions to select for microorganisms capable of degrading rice straw lignocellulose in its most recalcitrant form under industrially relevant conditions. Metatranscriptomes were sequenced from communities enriched under each temperature treatment. To determine specific thermo- and high-solids-tolerant lignocellulolytic enzymes potentially responsible for increased deconstruction in the thermophilic community, comparative metatranscriptomic analyses were performed to identify genes significantly overexpressed in the thermophilic community relative to the mesophilic community. Previous comparative metatranscriptomic studies have been performed to investigate lignocellulose degradation; however, they have focused on termite gut [10,11] and soil microbiota [12]. No work to date has utilized metatranscriptomics to identify new lignocellulolytic enzymes specifically active on biofuel feedstocks under industrial conditions. Furthermore, there is not yet a standard approach for determining statistical significance in differential expression results for microbial communities with largely differing structures. Such communities lack common genes, eliminating the ability to use transcript fold change as a metric to determine differential expression, gene by gene, across treatments. To address this issue, this study pooled gene expression by functional category, as indicated by Pfam annotations, since microbial communities performing similar tasks (such as biomass deconstruction) are likely to have overlapping functions even if they share no specific genes. The data analysis approaches presented in this study facilitated the discovery of glycoside hydrolases overexpressed under thermophilic conditions that may be useful for improving industrial enzymatic biomass deconstruction processes.

## Results

### Metatranscriptome metrics

**Figure 1:**
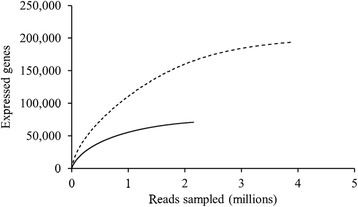
**Rarefaction curves describing number of expressed genes detected versus number of reads sampled for the mesophilic community (dashed line) and thermophilic community (solid line).**

Sequencing generated 68,754,440 reads for the mesophilic community and 50,014,968 reads for the thermophilic community (Table 1). Of these reads, 36.3% and 51.3% were filtered out as rRNA sequences from the mesophilic and thermophilic data sets, respectively. The remaining filtered reads were mapped to previously sequenced metagenomes from the same microbial communities [8]. 8.9% of mesophilic community reads were mapped to genes in the corresponding metagenome while 8.8% of reads were mapped to genes for the thermophilic community, indicating that many reads did not contain sufficiently unique sequence information to permit mapping to a single gene with confidence. Reads that mapped to intergenic or non-coding DNA were not included when determining these mapping percentages. 22.9% and 16.2% of genes were detected as expressed in the mesophilic and thermophilic communities, respectively, based on the fraction of genes in each metagenome that had at least one read mapped to them from the corresponding metatranscriptome. The total read count for genes within the lignocellulolytic glycoside hydrolase Pfams listed in Table 2 represented the size of the lignocellulolytic metatranscriptome for each community. Based on these values, expression of deconstructive glycoside hydrolases was estimated to constitute 0.16% of all gene expression in both the mesophilic and thermophilic communities. Rarefaction analysis showed clear asymptotes for both communities, suggesting that there was sufficient sequence coverage to detect most expressed genes (Figure 1). Size factors were calculated as 0.48 and 2.08 for the thermophilic and mesophilic communities, respectively, indicating approximately four times greater coverage of the mesophilic metatranscriptome.

**Table 1:** **Metatranscriptome sequencing and processing metrics**

	**Mesophilic community**	**Thermophilic community**
Total reads generated	68,754,440	50,014,968
mRNA reads	43,825,869	24,348,655
Mapped mRNA reads	3,916,829	2,153,529
Lignocellulolytic transcriptome reads	6,167	3,481

**Table 2:** **Protein families containing glycoside hydrolase (GH) or carbohydrate binding module (CBM) domains relevant to lignocellulose deconstruction that were targeted during metatranscriptome analysis**

**Protein family**	**Type**	**Family**	**Dominant types**
Pfam00150	GH	5	β-mannosidase, endo-β-1,4-glucanase, endo-β-1,4-mannosidase, endo-β-1,4-xylanase, β-1,4-cellobiosidase, β-1,3-mannanase, xyloglucan-specific endo-β-1,4-glucanase, exo-β-1,4-glucanase
Pfam00232	GH	1	β-glucosidase
Pfam00331	GH	10	Endo-β-1,4-xylanase, endo-β-1,3-xylanase
Pfam00457	GH	11	Xylanase
Pfam00722	GH	16	Endo-β-1,3-glucanase, endo-β-1,3(4)-glucanase, xyloglucanase
Pfam00759	GH	9	Endoglucanase, cellobiohydrolase, β-glucosidase
Pfam00840	GH	7	Endo-1,4-β-glucanase, cellobiohydrolase
Pfam00933	GH	3	β-glucosidase, 1,4-β-xylosidase, exo-1,3-1,4-glucanase, α-L-arabinofuranosidase
Pfam01270	GH	8	Cellulase, endo-β-1,4-xylanase, reducing-end xylose-releasing exo-oligoxylanase
Pfam01341	GH	6	Endoglucanase, cellobiohydrolase
Pfam01670	GH	12	Endoglucanase, xyloglucan hydrolase, β-1,3-1,4-glucanase
Pfam01915	GH	3C	β-glucosidase, β-1,4-xylosidase, exo-1,3-1,4-glucanase, α-L-arabinofuranosidase
Pfam02011	GH	48	Reducing end-acting cellobiohydrolase, endo-β-1,4-glucanase
Pfam02015	GH	45	Endoglucanase
Pfam02156	GH	26	β-mannanase, β-1,3-xylanase
Pfam03443	GH	61	Lytic polysaccharide monooxygenase
Pfam03648	GH	67 N	α-glucuronidase xylan, α-1,2-glucuronidase
Pfam03664	GH	62	α-L-arabinofuranosidase
Pfam04616	GH	43	β-xylosidase, α-L-arabinofuranosidase, arabinanase, xylanase
Pfam07477	GH	67C	α-glucuronidase, xylan, α-1,2-glucuronidase
Pfam07488	GH	67 M	α-glucuronidase, xylan, α-1,2-glucuronidase
Pfam00553	CBM	2	n/a
Pfam00734	CBM	1	n/a
Pfam00942	CBM	3	n/a
Pfam02013	CBM	10	n/a
Pfam02018	CBM	4, 9	n/a
Pfam03067	CBM	33	n/a
Pfam03422	CBM	6	n/a
Pfam03424	CBM	17, 28	n/a
Pfam03425	CBM	11	n/a
Pfam03426	CBM	15	n/a
Pfam09212	CBM	27	n/a
Pfam09478	CBM	49	n/a

### Composition of lignocellulolytic metatranscriptomes

Abundance data for sequences annotated with lignocellulolytic glycoside hydrolase (GH) Pfams (Table 2) show differing profiles in the GHs produced by enriched thermophilic and mesophilic communities (Table 3). The majority of cellulase expression in the thermophilic community corresponded to GH families 6, 9, and 48. Expression of these three GH families constituted 30% of the community’s lignocellulolytic metatranscriptome. Hemicellulase transcripts were observed from enzymes primarily in the GH families 10, 11, and 43 for the thermophilic community, with these GH families comprising 34.5% of the lignocellulolytic metatranscriptome. Expression of oligosaccharide-active enzymes from GH families 1 and 3 accounted for 23.2% of the thermophilic lignocellulolytic transcriptome.

**Table 3:** **Expression of lignocellulolytic glycoside hydrolase families in enriched mesophilic and thermophilic microbial communities**

	**% of lignocellulolytic GH transcriptome** ^*^	
**GH family**	**Thermophilic community**	**Mesophilic community**
1	10.9	3.9
3	12.3	28.8
5	1.8	9.6
6	16.1	1.4
7	1.4	0.0
8	0.7	1.4
9	4.4	3.0
10	12.3	4.6
11	13.8	4.4
12	1.3	0.2
16	2.2	9.4
26	0.1	2.1
43	8.4	24.4
45	0.0	0.1
48	9.6	0.5
61	2.8	0.1
62	0.9	0.3
67	1.2	5.8

*Values are given as percentages of the total number of normalized read counts mapped to lignocellulolytic glycoside hydrolase Pfams in the corresponding metagenome.

Expression of GH families that primarily have predicted cellulase activity (families 6, 7, 9, 45, and 48) and hemicellulase activity (families 10, 11, and 26) by the mesophilic community totaled 5.1% and 11.2% of the lignocellulolytic metatranscriptome, respectively. Expression of oligosaccharide-active GH family 3 enzymes accounted for 28.8% of the lignocellulolytic transcriptome. The remaining lignocellulolytic GH expression was primarily observed in GH families that span multiple activities. Expression of GH family 5 and 16 enzymes, which can have cellulase or hemicellulase activity, constituted 18.9% of the lignocellulolytic transcriptome, while GH family 43, which contains enzymes that act on hemicellulose and hemicellulose-derived oligosaccharides, accounted for 24.4% of the lignocellulolytic metatranscriptome.

### Phylogenetic classification of expressed lignocellulolytic enzymes

**Figure 2:**
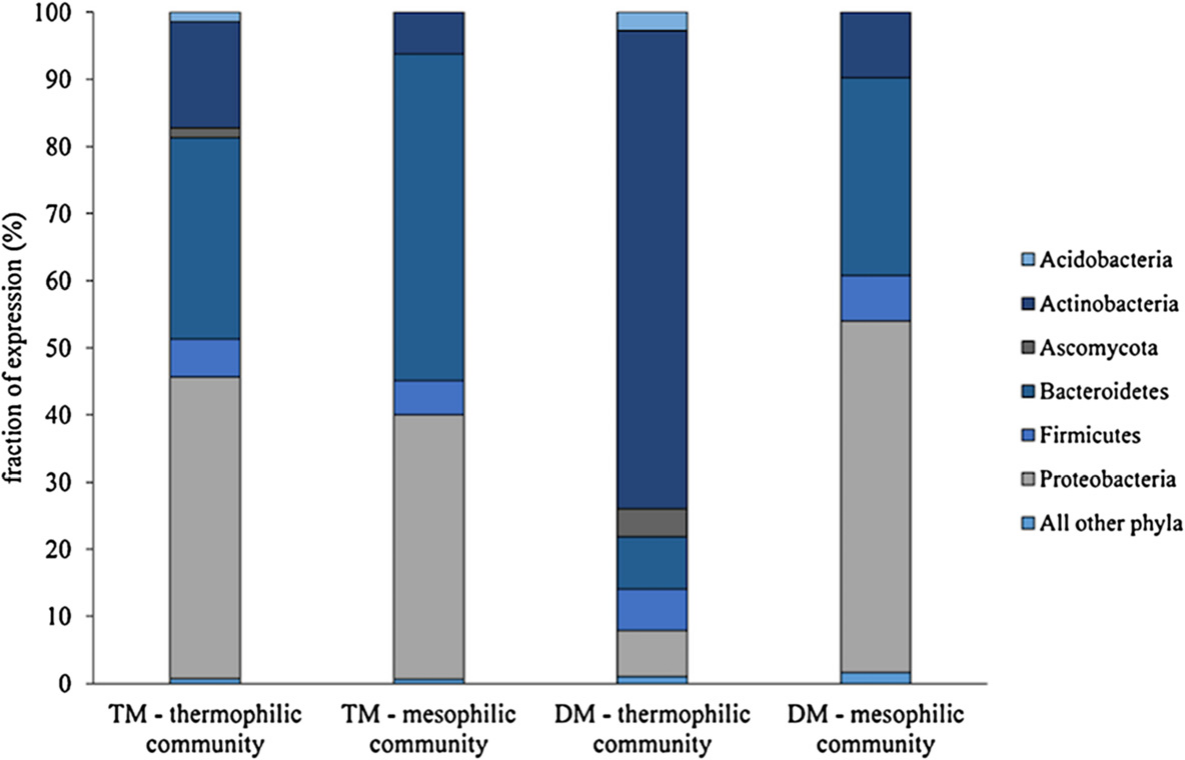
**Total gene expression and expression of glycoside hydrolase families relevant to lignocellulose deconstruction by phylum in thermophilic and mesophilic enriched communities.** TM - total metatranscriptome, all reads in metatranscriptome considered; DM - deconstructive metatranscriptome, only reads for deconstructive glycoside hydrolase Pfams considered.

Mapped metatranscriptome reads were coupled with phylogenetic binning data for corresponding metagenomes to determine the taxonomy of all mapped reads. Additional analysis focused solely on expressed enzymes with lignocellulolytic GH Pfam annotations. At the phylum level, total gene expression in both the thermophilic and mesophilic communities was predominantly by Proteobacteria and Bacteroidetes (Figure 2). Expression of the lignocellulolytic metatranscriptome was similarly dominated by Proteobacteria and Bacteroidetes in the mesophilic community. In contrast, the majority of the lignocellulolytic metatranscriptome was expressed by Actinobacteria in the thermophilic community (Figure 2). Bacteroidetes contributed to lignocellulolytic GH expression in both communities, although they played a more prominent role in the mesophilic community. For both communities, Firmicutes expressed a similar yet minor fraction of the total lignocellulolytic metatranscriptome. Fungi from the Ascomycota phylum contributed to lignocellulolytic GH expression in the thermophilic community, while fungal expression was not detected in the mesophilic community.

At the genus level, the prominence of Actinobacteria*-*expressed lignocellulolytic GH enzymes in the thermophilic community was primarily attributed to a single genus, *Micromonospora*. Expression of lignocellulolytic GH enzymes by *Micromonospora* spanned several activities, including endo- and exo-glucanases, hemicellulases, and oligosaccharide-active enzymes (Table 4). However, the majority of GH family 26, 43, and 67 expression, corresponding to hemicellulases, was from the *Niabella* and *Niastella* genera from phylum Bacteroidetes*.* Fungi from the genus *Chaetomium* expressed GH family 7, 16, and 61 enzymes. *Pseudoxanthomonas* from the Proteobacteria phylum expressed the predominate fraction of the GH family 8 enzymes in the thermophilic community. Alternately, *Pseudoxanthomonas* played a more prominent role in expression of the lignocellulolytic metatranscriptome of the mesophilic community. In particular, *Pseudoxanthomonas* was responsible for the majority of expression of GH family 8, 9, 10, 11, 43, and 67 enzymes. Similar to the thermophilic community, the Bacteroidetes genus *Niastella* contributed to expression of GH family 16 and 26 hemicellulose-active enzymes in the mesophilic community. However, in the mesophilic community *Niastella* also contributed to expression of GH family 5, 10, and 11 enzymes. Another Bacteroidetes genus, *Chryseobacterium*, uniquely featured in the mesophilic metatranscriptome as the most prominent source of GH family 3 enzyme expression.

**Table 4:** **Genera that express >50% of lignocellulolytic glycoside hydrolases in enriched thermophilic and mesophilic communities**

	**Thermophilic community**		**Mesophilic community**	
**GH family**	**Genus (Phylum)**	**% of transcripts for GH family**	**Genus (Phylum)**	**% of transcripts for GH family**
1	*Micromonospora* (A)	83.4	*Leifsonia* (A)	23.7
			*Cupriavidus* (P)	12.1
			*Bordetella* (P)	11.4
			*Pseudoxanthomonas* (P)	8.9
3	*Micromonospora* (A)	36.1	*Chyseobacterium* (B)	35.2
	*Pseudoxanthomonas* (P)	21.0	*Pseudoxanthomonas* (P)	23.9
5	*Micromonospora* (A)	57.7	*Niastella* (B)	16.8
			*Pseudoxanthomonas* (P)	16.5
			*Brevibacillus* (F)	15.8
			*Bordetella* (P)	10.9
6	*Micromonospora* (A)	93.8	*Planctomyces* (Pl)	35.3
			*Sphingobium* (P)	33.9
7	*Chaetomium* (As)	70.2	n/a	
8	*Pseudoxanthomonas* (P)	44.7	*Pseudoxanthomonas* (P)	69.3
	*Thermobacillus* (F)	40.0		
9	*Micromonospora* (A)	79.8	*Pseudoxanthomonas* (P)	45.8
			*Bordetella* (P)	20.8
10	*Micromonospora* (A)	65.5	*Pseudoxanthomonas* (P)	25.1
			*Niastella* (B)	20.1
			*Paenibacillus* (F)	17.4
11	*Micromonospora* (A)	94.7	*Pseudoxanthomonas* (P)	47.7
			*Niastella* (B)	25.1
12	*Micromonospora* (A)	86.7	*Leifsonia* (A)	73.6
16	*Chaeotomium* (As)	27.1	*Bordetella* (P)	29.2
	*Micromonospora* (A)	18.5	*Niastella* (B)	18.7
	*Niastella* (B)	17.8	*Chryseobacterium* (B)	17.0
26	*Niastella* (B)	65.2	*Leifsonia* (A)	24.9
			*Niastella* (B)	21.1
			*Brevibacillus* (F)	15.3
43	*Niabella* (B)	38.9	*Pseudoxanthomonas* (P)	34.3
	*Thermobacillus* (F)	23.9	*Bordetella* (P)	18.7
45	n/a		*Brevibacillus* (F)	100
48	*Micromonospora* (A)	100	*Bordetella* (P)	43.1
			*Sphingopyxis* (P)	22.1
61	*Chaetomium* (As)	49.0	*Pseudoxanthomons* (P)	100
		43.2		
	*Candidatus Solibacter* (Ac)			
62	*Micromonospora* (A)	46.0	*Niastella* (B)	100
	*Mycobacterium* (A)	40.5		
67	*Niabella* (B)	39.5	*Pseudoxanthomonas* (P)	50.7
	*Micromonospora* (A)	26.9		

Phyla are indicated as A, Actinobacteria; Ac, Acidobacteria; As, Ascomycota; B, Bacteroidetes; F, Firmicutes; P, Proteobacteria; Pl, Planctomycetes.

### Differential expression of lignocellulolytic enzymes between thermophilic and mesophilic communities

**Figure 3:**
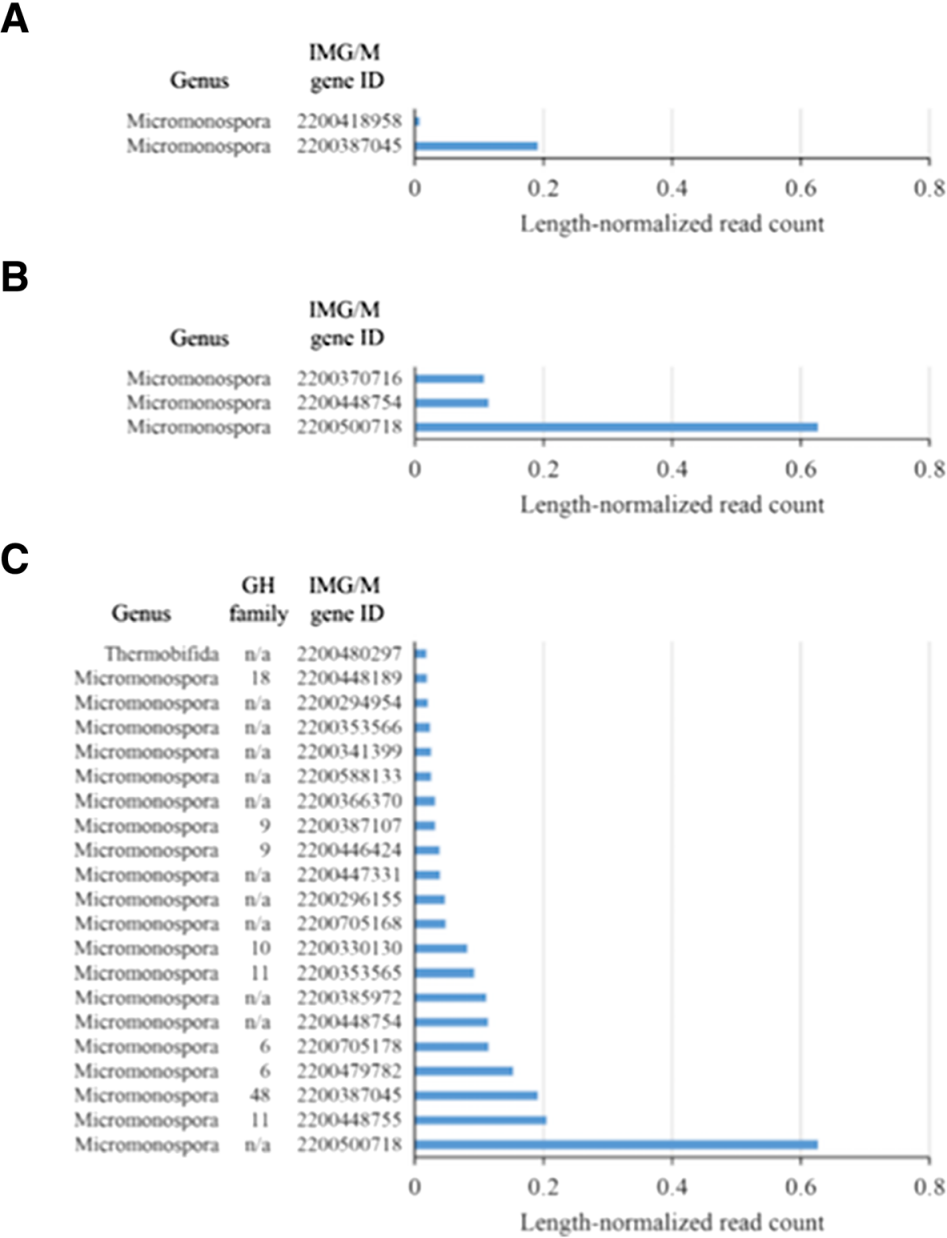
**Expression levels in the enriched thermophilic community for individual genes annotated as (A) Pfam02011 (glycoside hydrolase family 48), (B) Pfam03067 (carbohydrate binding module family 33), and (C) Pfam00553 (carbohydrate binding module family 2).** Genes listed account for ≥90% of all expression within a given Pfam.

The two methods utilized for determining differential expression of lignocellulolytic Pfams indicated that several GH and carbohydrate binding module (CBM) families relevant to lignocellulose deconstruction were significantly overexpressed in the thermophilic community (Table 5). Both methods concluded that there is evidence of significant overexpression of enzymes with GH family 48, CBM family 2, and CBM family 33 domains in the thermophilic community. Genes within these overexpressed Pfams were selected for further analysis. Examination of individual gene expression levels within each of these Pfams in the thermophilic community showed that Pfam overexpression in the thermophilic community can be attributed to the expression of a small number of genes (Figure 3). For genes annotated as GH family 48 (Pfam02011), 7 genes out of 12 total genes detected in the thermophilic metagenome were expressed (that is, had a read count ≥1), all of which belonged to genus *Micromonospora.* However, it was expression of a single *Micromonospora* GH family 48 gene (Joint Genome Institute Integrated Microbial Genomes with Microbiomes (IMG/M) gene ID 2200387045) in the thermophilic community that resulted in overexpression over the mesophilic community (Figure 3A). Similarly, 8 out of 17 genes with CBM family 33 (Pfam03067) annotation were expressed in the thermophilic community and all expressed genes were housed by *Micromonospora*. Likewise, expression of a single CBM family 33 gene (IMG/M gene ID 2200500718) was primarily responsible for elevated expression of the Pfam relative to the mesophilic community (Figure 3B). Both of these highly expressed genes also contained carbohydrate binding module family 2 domains (Pfam00553) (Figure 3C). As a result, expression of both of these genes contributed to the overexpression of genes encoding the CBM family 2 domain in the thermophilic community. However, additional enzymes containing the CBM family 2 domain were also highly expressed. These enzymes spanned other GH families, including families 6, 9, 10, and 11. Alignment of the amino acid sequence of the highly expressed GH family 48 enzyme against the National Center for Biotechnology Information (NCBI) non-redundant protein sequences database using the protein Basic Local Alignment Search Tool (BLAST) algorithm with the BLOSUM62 pair-score matrix [13] showed that the best match yielded only 76% identity with a GH family 48 enzyme from an uncultured bacterium [GenBank:AEM44250.1]. Alignment against GH family 48 enzymes from sequenced *Micromonospora* sp. yielded a maximum identity match of 63% (NCBI reference sequences YP_004083796.1 and YP_003837256.1). Both *Micromonospora* best matches corresponded to proteins predicted to be cellobiohydrolases. The GH family 48 gene discovered in the thermophilic community constitutes an open reading frame, and the length of the enzyme (970 amino acids) is similar to the length of the two most similar *Micromonospora* GH family 48 genes in the NCBI database (968 amino acids), suggesting that the sequence represents a complete gene. Similar alignment analysis of the CBM family 33 enzyme overexpressed in the thermophilic community showed a best match with 73% identity to a CBM family 33 protein (NCBI reference sequence YP_004406840.1) from *Verrucosispora maris*, a bacterium from the same family as *Micromonospora*. The best alignment to a sequenced *Micromonospora* CBM family 33 protein (NCBI reference sequence WP_007071991.1) resulted in 70% identity. The discovered gene encompasses an open reading frame, and its length of 363 amino acids is comparable to the 358 amino acid length of the most similar *Micromonospora* gene in the database, suggesting that the discovered sequence captures an intact gene.

**Table 5:** **Differential expression of protein families associated with lignocellulolytic activity in enriched thermophilic and mesophilic communities**

**Pfam**	**Name**	**Overexpressed in** ^**1**^	**Fold change over other community**	***P*** **-value, pseudo-Pfam comparison method** ^**4,5**^	**Adjusted** ***P*** **-value, DESEQ method** ^**5**^
Pfam00150	GH5	M	2.73	0.960	1.000
Pfam00232	GH1	T	9.33	0.333	0.331
Pfam00331	GH10	T	6.90	0.540	0.369
Pfam00457	GH11	T	7.53	0.109	0.369
Pfam00722	GH16	M	2.57	0.769	1.000
Pfam00759	GH9	T	4.79	0.542	0.569
Pfam00840	GH7	T	n/a^2^	0.291	0.256
Pfam00933	GH3	M	1.26	0.970	1.000
Pfam01270	GH8	T	1.28	0.975	1.000
Pfam01341	GH6	T	59.68	0.152	**0.018**
Pfam01670	GH12	T	20.49	0.623	0.466
Pfam01915	GH3C	M	1.39	0.864	1.000
Pfam02011	GH48	T	129.95	**0.105**	**0.012**
Pfam02015	GH45	M	n/a^2^	0.751	1.000
Pfam02156	GH26	M	7.94	0.890	0.878
Pfam03443	GH61	T	113.00	0.364	0.323
Pfam03648	GH67N	M	1.10	0.859	1.000
Pfam03664	GH62	T	42.87	0.282	**0.093**
Pfam04616	GH43	M	1.16	0.946	1.000
Pfam07477	GH67C	M	1.06	0.840	1.000
Pfam07488	GH67M	M	1.15	0.667	1.000
Pfam00553	CBM2	T	30.07	**0.097**	**0.012**
Pfam00734	CBM1	n/a^3^	n/a^3^	n/a^3^	n/a^3^
Pfam00942	CBM3	T	29.40	0.179	0.221
Pfam02013	CBM10	M	n/a^2^	0.485	**0.093**
Pfam02018	CBM4,9	M	1.47	0.247	1.000
Pfam03067	CBM33	T	49.61	**0.028**	**0.016**
Pfam03422	CBM6	M	2.31	0.930	1.000
Pfam03424	CBM17,28	M	n/a^2^	0.956	1.000
Pfam03425	CBM11	M	1.27	0.988	1.000
Pfam03426	CBM15	M	n/a^2^	0.656	1.000
Pfam09212	CBM27	n/a^3^	n/a^3^	n/a^3^	n/a^3^
Pfam09478	CBM49	n/a^3^	n/a^3^	n/a^3^	n/a^3^

^1^T, thermophilic community; M, mesophilic community.

^2^Fold change cannot be calculated because read count is 0 in one metatranscriptome.

^3^Pfam expression not detected in either metatranscriptome.

^4^ *P*-value is the mean from running the algorithm three times.

^5^Bold values indicate *P*-values ≤ to the critical value of 0.1.

## Discussion

Comparative metatranscriptomic analyses have offered new insight into how microbial communities respond to varying environmental conditions at a functional level [11,14,15]. These studies have demonstrated that communities with dissimilar gene contents can be compared on the basis of protein functional categories. For comparison of metatranscriptomes with low replicate numbers, random sampling of reads from the metatranscriptome has been used to create sub-metatranscriptomes that can be repeatedly compared to gauge the probability of observing differences in expression of functional categories between communities due to random chance [14,16]. In this way, one can assign statistical significance to observed differences in functional category expression levels. In the present work, a random sampling approach was used that compares expression of each functional category by considering the number of genes annotated to each functional category within a particular community. This differs from the prior technique in that statistical comparisons are made on the basis of randomly assembled groups of genes that mirror the number of genes within each functional category in the community data set rather than sampling a fixed number of genes randomly and relying on chance to capture genes from a functional category of interest. When comparing functional categories that have a small number of genes annotated to them relative to the number of genes in the metatranscriptome, this technique eliminates the risk of not capturing a particular functional category. This new approach was validated by showing that functional categories identified as differentially expressed between the thermophilic and mesophilic communities largely agree with those found by other methods developed for isolate comparative transcriptomics. In this study, only Pfams determined to have significant differential expression by both techniques were selected for further analysis. Other Pfams that register as significantly differentially expressed by only one of the methods should be interpreted with caution and warrant reevaluation as additional statistical methods are developed.

Previous researchers have coupled metatranscriptomic analysis with analysis of the corresponding metagenomes to complement expression data with additional information regarding gene content and taxa abundance in order to increase understanding of the microbial communities [15]. For the metatranscriptomes considered in the present work, previous metagenome studies have demonstrated that bacteria from genus *Micromonospora* are heavily enriched from a complex initial community following solid-state culture on rice straw under thermophilic conditions [8]. Moreover, metagenomic analysis of these enriched communities revealed that *Micromonospora* bacteria contain an array of genes coding lignocellulolytic enzymes, many containing CBM family 2 domains [8]. The metatranscriptomic data presented here suggest that the prominence of *Micromonospora* in the enriched thermophilic community is reflected in the active lignocellulose deconstructing community. Prior work has proposed *Micromonospora* species as potential lignocellulose degraders in the termite gut [17] and in rice straw compost [18], both high-solids environments. Additionally, several *Micromonospora* species have previously been observed in thermophilic compost [19]. The results presented here indicate that *Micromonospora*-derived deconstructive enzymes may also be active at high temperatures under high-solids conditions. Previous research has shown that the enriched thermophilic community was more active on rice straw compared to the enriched mesophilic community, as indicated by higher respiration rates during solid-state culture, suggesting that the thermophilic community had higher rates of polymer deconstruction and sugar utilization from the lignocellulosic biomass [8]. Moreover, measurements of endoglucanase and xylanase activities for enzymes extracted from enriched communities revealed that the thermophilic community exhibited increased levels of both activities compared to the mesophilic community [8]. These observations, combined with metatranscriptome data showing that *Micromonospora* dominates expression of lignocellulolytic enzymes in the thermophilic community, make this genus a promising source of lignocellulolytic enzymes for industrial high-solids deconstruction processes.

Specific *Micromonospora* genes within the Pfams overexpressed in the thermophilic community potentially code for novel enzymes, based on their similarity to existing genes in protein databases. These genes include an enzyme containing both GH family 48 and CBM family 2 domains. While exhibiting minimal cellulolytic activity on their own, enzymes from this GH family are known to enhance deconstruction of cellulose when combined with endoglucanases [20,21]. The presence of GH family 48 enzymes, some with CBM family 2 domains, has been noted previously in other cellulolytic *Actinobacteria* [21,22]. Furthermore, thermophilic microbial communities from compost have been shown to be rich in GH family 48 genes [23]. Prior study of the thermophilic community’s metagenome revealed that *Micromonospora* bacteria within the community harbored many putative cellobiohydrolases with CBM family 2 domains [8]. That only one of these genes is highly expressed in the thermophilic community may suggest it is particularly well suited to the particular feedstock, temperature, and moisture level used for enrichment.

The second overexpressed *Micromonospora* gene coded for an enzyme with CBM family 33 and family 2 domains. CBM family 33 enzymes have increasingly garnered interest due to their recently discovered lytic polysaccharide monooxygenase activity. Although the exact mechanism is not completely understood, certain CBM family 33 enzymes are able to cleave cellulose chains in their crystalline form, increasing the amount of cellulose chain ends [24]. It is thought that this action synergistically enhances cellulose deconstruction by making cellulose more accessible to other cellulases. Research has shown that combining CBM family 33 enzymes with other cellulases increases cellulolytic activity [25]. Like the overexpressed GH family 48 enzymes, the prominence of the overexpressed *Micromonospora* CBM family 33 in the thermophilic community indicates that it is active under industrial deconstruction conditions. Furthermore, as both overexpressed genes contain CBM family 2 domains, this CBM may be effective at binding cellulose under thermophilic, high-solids conditions and may be useful for engineering other cellulolytic enzymes tolerant to these conditions. Notably, past work has shown that GH family 48 and CBM family 33 enzymes containing CBM family 2 were also prominent in the secretomes of *Streptomyces* grown aerobically on cellulose or switchgrass [26]. These enzymes may represent a lignocellulolytic mechanism unique to aerobic Actinobacteria. Considering the overexpression of these genes and the enhanced deconstructive activity of the thermophilic community over the mesophilic community, these enzymes warrant additional study to better characterize their activity and determine their benefit to industrial lignocellulose deconstruction for biofuel production.

## Conclusions

Analyzing metatranscriptomes from microbial communities enriched under conditions relevant to industrial deconstruction of lignocellulosic feedstocks for biofuel production is a powerful technique for discovering potentially robust lignocellulolytic enzymes. While the enrichment culture strategy may lead to communities that differ drastically in composition based on treatment, with few shared genes between them, differential expression analyses can still be performed by considering expression of gene functional categories, such as Pfams. Using this approach in conjunction with metagenomic data, candidate cellulase and polysaccharide monooxygenase genes from significantly overexpressed Pfams in the thermophilic community were identified. Their unique presence and high expression level in the thermophilic community make them promising candidates for improving deconstruction processes under high-temperature and high-solids conditions.

## Materials and Methods

### Enrichment cultures

Rice straw (*Oryza sativa L.*, California rice M206) was collected, dried, milled, and stored as previously described [27]. High-solids enrichment cultures under mesophilic and thermophilic conditions were performed as previously described [8]. In brief, rice straw was inoculated with green waste compost to achieve 0.1 g compost/g mixture (dry weight basis) and then wetted to 0.8 g water/g mixture (fresh weight basis) with carbon-free minimal media [7]. The wetted biomass was allowed to equilibrate overnight at 4°C. Solid-state culture bioreactors with a 200-ml capacity [28] were loaded with 5 to 10 g (dry weight) of wetted material and aerated at 10 ml/min. For the mesophilic enrichment, one reactor was incubated at 35°C. For the thermophilic enrichment, one reactor underwent an initial temperature ramp from 35°C to 55°C by increasing the temperature by 5°C every 6 hours. The temperature was then held at 55°C for the remainder of the incubation. Water was added to the reactors twice weekly to maintain a constant moisture content during incubation. Cultures were passaged weekly by inoculating freshly wetted rice straw with cultured biomass to achieve 0.1 g cultured biomass/g mixture (dry weight basis). The cultured biomass underwent RNA extraction four weeks after the initial inoculation.

### RNA preparation

Samples were stabilized by adding an excess of LifeGuard Soil Preservation Solution (MoBio Laboratories, Inc., Carlsbad, CA) to 2-g aliquots of biomass. RNA was extracted from stabilized samples using an RNA PowerSoil total RNA isolation kit (MoBio Laboratories, Inc.) according to the manufacturer’s instructions with 25 μl of 2-mercaptoethanol added to each sample during the bead solution addition step. Eluates with isolated RNA were processed using an RNeasy mini kit (Qiagen, Venlo, Netherlands). For each sample, 100 μl of eluate was combined with 350 μl of buffer RLT from the kit and 3.5 μl of 2-mercaptoethanol, mixed vigorously, and combined with 250 μl of 100% ethanol. The entire volume of solution was loaded onto an RNeasy column and centrifuged for 30 s at ≥8,000 × g. In lieu of the wash step, digestion of genomic DNA in samples was performed using RNase-free DNase (Qiagen), as described in the manufacturer’s protocol for the RNeasy kit. Following DNA digestion, samples were processed as described in the RNA cleanup portion of the manufacturer’s instructions. An additional digestion was performed using a TURBO DNA-free kit (Applied Biosystems, Carlsbad, CA) to remove residual genomic DNA. Digestions were performed in a 200-μl reaction volume with 20 to 30 μg RNA according to the manufacturer’s protocol with the following exceptions: DNase loading was increased to 0.3 μl DNase solution/μg RNA and the final centrifugation step to remove DNase inactivation reagent was increased to 10 min. The treated RNA was concentrated by adding 3× volume of 100% ethanol, incubating at -20°C for at least 2 hours, washing with 70% ethanol, and resuspending the dried pelleted RNA in diethylpyrocarbonate (DEPC)-treated water. The processed RNA was stored at -80°C.

A MICROBExpress Bacterial mRNA Enrichment Kit (Ambion, Carlsbad, CA) was used to enrich mRNA from 10 μg of extracted RNA. Ambion Fragmentation Reagents were used to fragment mRNA. cDNA was generated from fragmented mRNA using a SuperScript Double Stranded cDNA Synthesis Kit (Invitrogen, Carlsbad, CA) according to the manufacturer’s guidelines. Random hexamers were used as primers during strand synthesis. dNTP mix with dTTP substituted with dUTP was used during second strand synthesis. The resultant double-stranded cDNA was processed using a TruSeq DNA Sample Prep Kit (Illumina, San Diego, CA) to polish fragment ends, add A-tails, and ligate TruSeq adapters. Second strands were removed from processed cDNA through digestion of dUTP with AmpErase Urasil N-glycosylase (Applied Biosystems, Carlsbad, CA). cDNA was then enriched via 10 cycles of PCR with Illumina TruSeq primers.

### cDNA sequencing

cDNA sequencing was conducted using the Joint Genome Institute’s standard cDNA sequencing pipeline for the Genome Analyzer platform (Illumina). In brief, fragmented cDNA was fixed to a flow cell, and clusters were generated using a Paired-End Cluster Generation Kit v4 (Illumina). The first set of reads was generated from clusters using the Illumina Genome Analyzer and 36-cycle Sequencing Kit v4 (Illumina) according to the manufacturer’s instructions. Following the first run, clusters were resynthesized with the Paired-End Cluster Generation Kit v4, and paired-end reads were obtained using a second Genome Analyzer run. A read length of 151 bp was used for both runs.

### Data analysis

Metatranscriptome reads were filtered to identify rRNA sequences by using the HMMsearch command in HMMER [29] to align the reads against the Rfam [30], RDP [31], and NCBI [32] databases. For a read to be called as an rRNA sequence, a threshold of ≥30% identity between the read and the reference sequence was used and at least 70% of the read must have aligned to the reference sequence. Reads with alignment to rRNA sequences were removed from the data set to isolate mRNA sequences. Filtered reads were mapped to their corresponding metagenomes using a custom program developed by the US Department of Energy’s Joint Genome Institute that uses the Burrows-Wheeler Aligner [33] to align reads against metagenomic contigs and log the number of reads within each annotated gene. A read was considered to align with a gene if the midpoint of the read fell within the gene boundaries. The metagenomes used for mapping were sequenced, annotated, and phylogenetically binned previously [8] and are accessible through the Joint Genome Institute’s Integrated Microbial Genomes with Microbiomes (IMG/M) portal (https://img.jgi.doe.gov/cgi-bin/m/main.cgi) under taxon object IDs 2162886009 and 2162886010 for the mesophilic and thermophilic community metagenomes, respectively. PAST software [34] was used to perform rarefaction analysis on mapped read counts to determine the adequacy of sequence coverage for capturing expressed genes.

The metatranscriptomes were compared to determine differential expression of genes between microbial communities. Prior to comparison, the read count mapped to each gene was normalized by dividing by the gene length. Since the phylogenetic composition of each community was known to be different with few genes in common [8], differential expression analysis was performed on the basis of expression of Pfam functional categories [35]. For a comparison of two metatranscriptomes, *A* and *B*, each with a corresponding metagenome, normalized read counts for all genes were collated by Pfam annotation such that

1 $$ {P}_{i,A}=\frac{p_{i,A}}{S_A}=\frac{\left({\displaystyle {\sum}_{j=1}^{n_{i,A}}{g}_{j,i,A}/{l}_{j,i,A}}\right)}{S_A} $$

where *P*_*i,A*_ is the sum of all normalized read counts for genes with annotation to Pfam *i* in metatranscriptome *A*, *p*_*i,A*_ is the sum of length-normalized read counts for all genes with annotation to Pfam *i* in metatranscriptome *A, n*_*i,A*_ is the number of genes in metatranscriptome *A* with annotation to Pfam *i*, *g*_*j,i,A*_ is the read count for the *j*th gene with annotation to Pfam *i* in metatranscriptome *A*, *l*_*j,i,A*_ is the length of the *j*th gene with annotation to Pfam *i* in metatranscriptome *A*, and *S*_*A*_ is a size factor related to the sequencing depth of metatranscriptome *A* meant to normalize for differences in sequence coverage between metatranscriptomes *A* and *B*. Size factors were calculated using the method described by Anders and Huber [36]:

2 $$ {S}_A= median\left(\frac{p_{i,A}}{{\left({p}_i{{}_{,}}_A{p}_i{{}_{,}}_B\right)}^{1/2}}\right) $$

where for each metatranscriptome, the ratio of each Pfam’s length-normalized read count, *p*_*i,A*_ to the geometric mean of length-normalized read counts across all metatranscriptomes sampled is calculated and the median value is taken as the size factor for that metatranscriptome*. P*_*i,B*_ can be similarly calculated for Pfams in metatranscriptome *B.* Genes with multiple Pfam annotations were represented separately for each Pfam category. Genes lacking a Pfam annotation were discarded prior to collation.

For a given Pfam*,* the differential expression between metatranscriptomes *A* and *B, D*_*i*_, is described by *D*_*i*_ *= P*_*i,A*_*-P*_*i,B*_*.* The statistical significance of any observed *D*_*i*_ value was determined by evaluating the probability of observing a difference value ≥ *D*_*i*_ for two groups of randomly selected genes of sizes *n*_*i,A*_ and *n*_*i,B*_*.* To generate these random gene groupings for a given Pfam, referred to as pseudo-Pfams from here onward, a Matlab (version 7.4.0.739, MathWorks, Natick, MA) script was used to randomly select *n*_*i,A*_ genes with a Pfam annotation and non-zero read count from metratranscriptome *A* and sum their normalized read counts. Similarly, normalized read counts of *n*_*i,B*_ random genes were chosen and summed for metatranscriptome *B*. The difference in normalized read counts for the two pseudo-Pfams, *D*_*i,pseudo*_, was then calculated. This process of generating *D*_*i,pseudo*_ values was repeated 10,000 times for each Pfam to create a probability distribution of difference values that arise due to chance. The observed values of *D*_*i*_ were compared to corresponding probability distributions to determine the probability of obtaining the observed result by chance (the *P*-value). This approach was used to specifically analyze Pfams relevant to lignocellulose deconstruction (Table 2). Since multiple comparisons were conducted, the *P*-values for individual comparisons would normally be adjusted to yield a target family-wise error rate for false positives. However, given the goal of this study - to discover new enzymes for high-solids, high-temperature lignocellulose deconstruction - false negatives were deemed more undesirable than false positives, as false positives can be filtered out via later experimentation to measure enzyme activity in isolation while false negatives would be lost prior to further study. As a result, the family-wise error rate was determined to be less critical than that for individual hypotheses.

The method developed by Anders and Huber [36] for analyzing isolate transcriptomes was adapted and used as an additional technique to determine differentially expressed Pfams between communities. The method was performed using the DESEQ program implemented in R (version 2.15.3, The R Foundation for Statistical Computing, Vienna, Austria). In brief, read counts for each Pfam listed in Table 2 were used to estimate the mean and variance of a negative binomial distribution, which were then used to test the null hypothesis that read counts for a given Pfam do not differ between the two communities. As only one metatranscriptome was obtained from each community, the Pfam read counts from both communities were temporarily pooled and treated as duplicate samples in order to estimate the read count variance. This approach assumed that most Pfams were not differentially expressed between the communities. If this assumption is invalid, variance values will be overestimated and the probability of obtaining false negatives will increase, limiting detection of differential expression to Pfams with drastic differences in expression between the communities [36]. *P*-values from DESEQ analysis were adjusted using the Benjamini-Hochberg method [37] to account for multiple comparisons and provide a more stringent determination of differentially expressed Pfams compared to the previously described method. A false discovery rate of 0.10 was used to determine significance for both techniques.

### Data archiving

Metatranscriptome raw reads and mapped read counts are archived on IMG/M. These data can be accessed via the Joint Genome Institute portal under their corresponding metagenomes on IMG/M, which are listed as taxon object IDs 2162886009 and 2162886010 for the mesophilic and thermophilic communities, respectively.

## Abbreviations

BLAST: Basic Local Alignment Search Tool
CBM: carbohydrate binding module
DM: deconstructive metatranscriptome
GH: glycoside hydrolase
IMG/M: Integrated Microbial Genomes with Microbiomes
M: mesophilic community
NCBI: National Center of Biotechnoloy Information
T: thermophilic community
TM: total metatranscriptome
